# Diuresis and α-glucosidase inhibition by erythritol in *Aedes aegypti* (Diptera: Culicidae) and viability for efficacy against mosquitoes

**DOI:** 10.1186/s13071-024-06169-w

**Published:** 2024-02-20

**Authors:** Irvane E. Nelson, Kobi A. Baker, Ary Faraji, Gregory S. White, Christopher S. Bibbs

**Affiliations:** 1 Salt Lake City Mosquito Abatement District, 2215 North 2200 West, Salt Lake City, UT 84116 USA; 2 College of Science, Science Research Initiative (SRI), University of Utah, 1390 Presidents Circle, Crocker Science Center, rm 310, Salt Lake City, UT 84112 USA

**Keywords:** Starvation, Sugar alcohol, Excretion, Toxicity, Sugar meals

## Abstract

**Background:**

Sugar alcohols, such as erythritol, are low-impact candidates for attractive toxic sugar baits (ATSB) to kill mosquitoes. To determine whether erythritol has a viable future in ATSB formulations, a suite of assays was conducted to diagnose toxicity mechanisms and starvation effects on mortality in *Aedes aegypti* (L.) as a model system.

**Methods:**

We measured general carbohydrate load, glucosidase levels, and free glucose in intoxicated adult mosquitoes to observe whether sugar digestion was impaired. We assayed the effects of sugar combinations with erythritol on larvae and adults. To measure erythritol effects when mosquitoes were not resource-deprived, additional assays manipulated the prior starvation status.

**Results:**

Up to 50,000 ppm of erythritol in water had no effect on larvae within 72 h, but an ammonia spike indicated diuresis in larvae as early as 4 h (*F*_8,44_ = 22.50, *P* < 0.0001) after sucrose/erythritol combinations were added. Adult consumption of erythritol was diuretic regardless of the sugar pairing, while sucrose and erythritol together generated above 80% mortality (*F*_2,273_ = 33.30, *P* < 0.0001) alongside triple the normal excretion (*F*_5,78_ = 26.80, *P* < 0.0004). Glucose and fructose paired individually with erythritol had less mortality, but still double the fecal excretion. When ingesting erythritol-laced meals, less sugar was detected in mosquitoes as compared to after sucrose meals (*χ*^2^ = 12.54, *df* = 1, *P* = 0.0004).

**Conclusions:**

Data showed that erythritol is a linear competitive inhibitor of α-glucosidase, marking it as a novel class of insecticide in the current research climate. However, the efficacy on larvae was null and not persistent in adult mosquitoes when compared across various starvation levels. Despite significant diuresis, the combined effects from erythritol are not acute enough for vector control programs considering ATSB against mosquitoes.

**Graphical Abstract:**

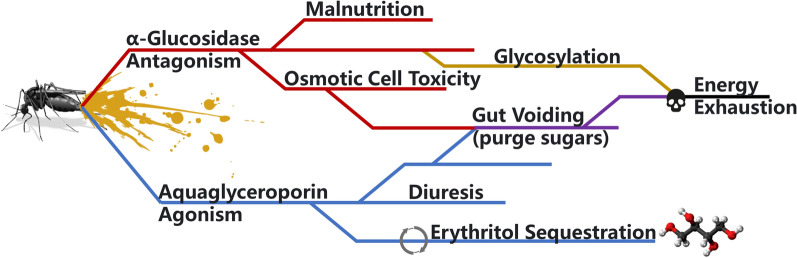

## Background

Erythritol, a sugar alcohol and food-grade artificial sweetener, has been observed in other animals to substitute glucose in the catalytic binding pocket of α-glucosidase [1]. Erythritolic inhibition in α-glucosidase has been directly measured once before in true bugs [2], and the sugar alcohol has since become a heavily discussed topic for the potential to kill mosquitoes [3], fruit flies [4], vinegar flies [5], house flies [6], ants [7], and psyllids [8]. Mosquitoes and other flies have been the focal pests in erythritol studies [3, 9–11]. Evidently, erythritol is also unique amongst other polyols, with competing molecules being largely ineffective in the aforementioned fly groups [4, 6, 9, 10, 12]. Despite what is already known, it is not clear whether erythritol is a linear competitive inhibitor of all α-glucosidases, or whether there are any secondary interactions that might improve toxicity.

For example, not all α-glucosidase hydrolases in mosquitoes or their larval stages are well characterized [13]. In other α-glucosidase systems, the Cqm1, a receptor site of *Lysinibacillus sphaericus* [14], and the homologue Agm3 as receptor for *Bacillus thuringiensis* (*Bt*) [15], have been linked with the necessary activation steps for Cry-toxin substrates and subsequent toxicity. Co-ingestion of erythritol and *Bt* could possibly reduce *Bt* gut binding affinity by competing with α-glucosidase, yet empirically erythritol and *Bt* co-ingestion in mosquitoes was only additive in efficacy [11]. It is possible that the reduction in available enzymes is rather small, or that erythritol is actually selective on certain α-glucosidase, or that redundant pathways may exist that allow recovery. It is also possible that the adult test system, which is not technically the target for *Bt* products, is an inappropriate test system. However, mosquito larvae, the most effective life stage for *Bt* toxins, were also susceptible to erythritol at a 5% concentration in water, or 50,000 parts per million (ppm) [3]. Operationally a 50,000-ppm treatment is unsustainably high for field use as a larvicide. But lower concentrations have not yet been tested. Unfortunately, no other observations were made for larval toxicity [3] to help unravel efficacy determinations between larval and adult mosquitoes. Larval mosquitoes should, therefore, be included in mechanistic studies in order to find conserved outcomes.

Recapitulating the available information, even though α-glucosidase is a putative target site for erythritol, it does not specifically answer why the insect dies and whether these effects are reliable for use in mosquito control programs. Erythritol was hypothesized to disrupt protein glycosylation in *Aedes aegypti* (L.), the yellow fever mosquito [9], to which the implicated mannose-1-phosphate guanylyltransferases are also known to be able to metabolize fructose [16]. Additionally, when vinegar flies ingested erythritol-laced sucrose meals, an increased solute load was measured in intoxicated flies [5, 12, 17]. Despite the osmotic stress, the authors attributed mortality to depleted glycogen [5, 12, 17]. But disparate observations in house flies and fruit flies described fatal regurgitation of gut contents [4, 6]. Interestingly, regurgitation severity was linked to co-ingestion with sucrose, though no other nutritive sugars were attempted [4]. Mosquito mortality also is improved when erythritol is co-ingested with sucrose [3, 11], even though there have been no observations to date that mosquitoes are regurgitating as a sign of toxicity from erythritol. It was also not clear in a prior study [11] whether the erythritol treatment was fatally diuretic, such as in house flies [6]. There are conflicting explanations across fly taxa about how erythritol functions within pest biology, despite an already healthy interest in using erythritol for attractive-toxic sugar baits (ATSB) [9, 11].

Regardless of its use as a larvicide or adulticide, we were interested in understanding the underlying cause of death from erythritol toxicity using *Ae. aegypti* as a model. Practitioners need to understand the mechanisms behind mortality to inform good practices for using erythritol, or similar ingredients. Erythritol may interact with glucosidase expression in mosquitoes [9, 11]. The efficacy of erythritol co-ingested with other sugars can also improve ATSB formulations [3, 4]. Alternatively, fatal diuresis [4, 6] may be a connected or parallel sign of toxicity. And finally, malnutrition [5, 12, 17] has been hypothesized to drive mortality in other flies. By exploring the above-listed items, we can make a reasonable decision on how erythritol can be formulated in ATSB (e.g., with other active ingredients, with specific sugars, in sublethal cocktails) and whether mosquito control operations should focus on this ingredient. The addition of a new active ingredient for vector control programs could prove crucial in combating mosquitoes and mosquito-borne pathogens for better public health protection.

## Methods

The 1952 Orlando strain (ORL) *Ae. aegypti* were reared in laboratory colonies at the Salt Lake City Mosquito Abatement District. Mosquito larvae were reared in collection trays, and adult flight cages were kept at consistent environmental conditions of 27 ± 1 °C temperature and 70 ± 5% relative humidity. Adults were fed 10% sucrose solution ad libitum during regular maintenance and deprived of sugar, but not water, 24 h prior to bioassays. Adults were between 5 and 10 days old, with opportunities to mate but not to blood-fed. Larvae used were at the third-instar stage.

### Cage bioassays

Larval bioassays were conducted in 250 ml of reverse osmosis (RO) water in 473 ml (16 oz) paper cups (760SOUP12WB, Choice Foodservice Equipment Company, Layton, UT, USA). Each cup contained 15 third-instar larvae of *Ae. aegypti.* Larvae were fed 0.5 ml of 4% bovine liver powder in water solution. Sucrose, erythritol, and a 1:1 ratio of sucrose/erythritol treatments were measured via an analytical balance in weighing boats and sprinkled evenly across the surface of the water in additions of 25 mg (100 ppm), 500 mg (2000 ppm), or 12.5 g (50,000 ppm). Feeding controls consisted of larvae only fed 0.5 ml of 4% bovine liver powder in water solution. Starvation controls consisted of larvae with no food or treatment, to assess starvation stress on measurements. Negative controls were fed sucrose in addition to the food slurry. All handling groups (treatments and controls) were replicated five times each. Larvae were observed over a period of 72 h, with mortality recorded every 24 h. A water sample was performed after the first 4 h and again at 24 h using a nitrogen test kit to look for excretory products that would signal diuresis, i.e. ammonia, nitrates, and nitrites (API Freshwater Master Test Kit, Mars Fishcare North America, Inc., Chalfont, PA, USA).

Adult bioassays were conducted using varying combinations of sucrose (Great Value Sugar, Walmart Inc., Bentonville, AR, USA), fructose (Modernist Pantry, LLC, Eliot, ME, USA), glucose (dextrose, Modernist Pantry, LLC, Eliot, ME, USA), and erythritol (Pyure Organic Erythritol, Pyure Brands, Naples, FL, USA). Trials were prepared in the same style of cup as used in larval bioassays, but with the addition of a fine mesh secured to the top of the cup with a rubber band. Holes were added to the sides of each cup to allow aspiration of mosquitoes and seating a 2 ml plastic microcentrifuge tube (2.0 ml Eppendorf Safe-Lock Tube, Eppendorf, Hamburg, Germany) filled with treatments. A small piece of cotton plugged the tube and allowed the solution to wick into the bioassay cup. A total of 18 ± 5 adult pre-starved *Ae. aegypti* were aspirated into each cup. The aspiration slot was then plugged with an additional cotton ball that was moistened daily with RO water to allow adequate hydration and humidity.

Treatments were combined with orange food-grade dye (McCormick & Company, Inc., Baltimore, MD, USA) in the following groupings: 10% sucrose; 5% sucrose; 5% glucose + 5% fructose; 2.5% glucose + 2.5% fructose + 5% erythritol; 5% fructose + 5% erythritol; 5% glucose + 5% erythritol; and 5% sucrose + 5% erythritol. All trials were conducted as no-choice bioassays and were stored in a Thermo Scientific 3920 Large Capacity Environmental Chamber kept at a constant 27° C, and 12:12 (light/dark). A tray of 2500 ml of RO water was nested in the chamber to generate 75% RH. All aforementioned groupings were assayed 12 times each. Cups were checked every 24 h for mortality over three consecutive days. Plain sucrose meals formed a natural baseline for excretion stress [18]; therefore, after the 3-day assays, any surviving adults were disposed of and diuretic response was measure through fecal droplet counts under a light dissection scope [19].

An additional set of experiments was performed to show the effect of nutrition level on mortality caused by erythritol. In all test groups in this assay, mosquitoes were fed in a no-choice assay using undyed 5% erythritol + 5% sucrose. Treatments varied by using six procedures: T1 = no pre-starvation period but with continuous access to toxicant; T2 = no pre-starvation period and toxicant replaced with 10% sucrose after 24 h of exposure; T3 = a 72 h period of only 3% sucrose and toxicant replaced with 10% sucrose after 24 h of exposure; T4 = a 24-h pre-starvation period with toxicant replaced with 10% sucrose after 24 h of exposure; T5 = a 72-h period of only 3% sucrose followed by continuous access to toxicant; T6 = a 24-h pre-starvation period and continuous access to toxicant. All mosquitoes were monitored for 72 h total and all other bioassay conditions were identical to the aforementioned trials. All groups were replicated 12 times.

### Reagent studies

Reagent studies were conducted to measure three portions in the sugar digestion process. Detection of α-glucosidase was used to estimate whether the mosquito was attempting to digest the sugars (more enzyme = more attempted digestion). Cold anthrone tests were used to detect the presence or absence of ingested sugars, which is also affected by excretion before the mosquitoes have a chance to fully digest. Glucose oxidase assays were used to measure the digestion product glucose, which is also an estimate of the biological likelihood of starvation. Generally, cages of 50 female, non-blood-fed mosquitoes were pre-starved for 24 h and given access to non-dyed treatments of either RO water, 5% sucrose, 10% sucrose, 5% erythritol, 10% erythritol, or 5% erythritol + 5% sucrose for 24 additional hours before assays. Fed mosquitoes were anesthetized at −80 °C and were randomly selected among those with at least partially distended abdomens and set aside for processing. Mosquitoes were sorted individually into 1.5 ml centrifuge tubes (C3017 Quik-Snap, Alkali Scientific, Inc., Ft. Lauderdale, FL, USA). Samples were covered with 50 µl of phosphate-buffered saline (pH 7.4 Gibco 10010-023, Fisher Scientific International, Inc., Hampton, NH, USA) and vortexed for 1 min with a homogenization bead. Homogenate was then centrifuged at 12,000 RPM for 5 min, after which 30 µl of reactants were transferred into associated reagent treatment groups.

For general carbohydrate detection, cold anthrone reactions were prepared according to Nunes et al. [20], with mosquitoes handled using procedures from Gu et al. [21]. Briefly, anthrone mixtures were prepared by adding technical-grade H_2_SO_4_ (Hi Valley Chemical, Inc., Centerville, UT, USA) to RO water until at 70% concentration was obtained. Then, 25 mg of anthrone (ANTH25G, Chemsavers, Inc., Bluefield, VA, USA) was added to 50 ml of the diluted acid. Prepared anthrone mixtures were cooled to room temperature before use. These reactions measure breakdown products of complex sugars, but are not specific to given monosaccharides. Therefore, standard curves were prepared using stock solutions of sucrose ranging from 200 µg/ml down to 0 µg/ml. With samples prepared as before, four replicates were conducted whereby 20 µl of mosquito reactants was transferred with a multichannel pipette and mixed in a 96-well plate with 200 µl of anthrone mixture before incubating at room temperature for 45 min. Spectra absorbance was measured at an initial reading of 620 nm, with sample data plotted against the standard curve for the associated replicate to derive a mosquito sucrose estimate.

Activity for α-glucosidase was monitored using an enzyme kit (MAK123-1KT, Sigma-Aldrich, Inc., St. Louis, MO, USA). The kit uses *p*-nitrophenyl-α-d-glucopyranoside, which is hydrolyzed specifically by α-glucosidase into a yellow-colored product with maximal spectra absorbance at 405 nm. The rate of the reaction is directly proportional to the enzyme activity. This results in a greater difference between initial and final absorption values when measured in a spectrophotometer. Assay reagents and mix preparations were conducted according to product instructions across six replicates of mosquitoes. Using a single-channel pipette, 20 µl of homogenized mosquito reactants was transferred and mixed into individual wells of a 96-well plate (24-301 Olympus 96-well plate, Genesee Scientific Co., Morrisville, NC, USA) such that each mosquito was isolated in an individually labeled well. Using a spectrophotometer (Synergy HTX, BioTek Instruments, Winooski, VT, USA), initial absorbance was measured at 405 nm (A405) and again every 5 min for a total runtime of 25 min while incubated at 37 °C.

Free glucose measurements were performed using a glucose reaction kit (A22189 Amplex Red Glucose Kit, Thermo Fisher Scientific, Inc., Waltham, MA, USA). As before, assay reagents and mix preparations were conducted according to product instructions; four replicates were conducted. Standard curves were prepared using stock solutions of sucrose ranging from 200 μg/ml down to 0 µg/ml. For glucose measurements, 10 µl of reactant, 40 µl of additional buffer, and 50 µl of assay mixture were combined and incubated at room temperature for 30 min. Spectra absorbance was measured at an initial reading of 560 nm, with sample data plotted against the standard curve for the associated replicate to derive a mosquito glucose estimate.

### Data analysis

Data were analyzed using R statistical software (v.4.2.1, R Foundation for Statistical Computing, Vienna, Austria) via RStudio (v. 3.3.0, RStudio PBC, Boston, MA, USA). Mortality for the sugar combination and starvation bioassays was analyzed using a repeated-measures analysis of variance (ANOVA) between time and treatment factors. Fecal droplets were adjusted to the female sample size (droplets/n) and compared by treatment to the 72-h mortality using a negative binomial generalized linear model (GLM), whereby 72-h mortality was the dependent variable, sugar combination as the independent variable, fecal droplets as a covariate, and with an additional interaction term of treatment by fecal. Differences between groups were analyzed by ANOVA and Tukey’s honestly significant difference test (HSD). Absorbance spectroscopy data from the glucosidase assays were converted using Beer’s law (Beer-Lambert equation). Anthrone and glucose results were corrected for the RO water controls and converted to sugar estimates from the standard curve results. Larval ammonia data were analyzed with ANOVA and Tukey’s HSD. Converted anthrone/glucosidase/glucose data were analyzed with Kruskal–Wallis and Wilcoxon signed-rank test due to unequal variance between groups.

## Results

**Fig. 1:**
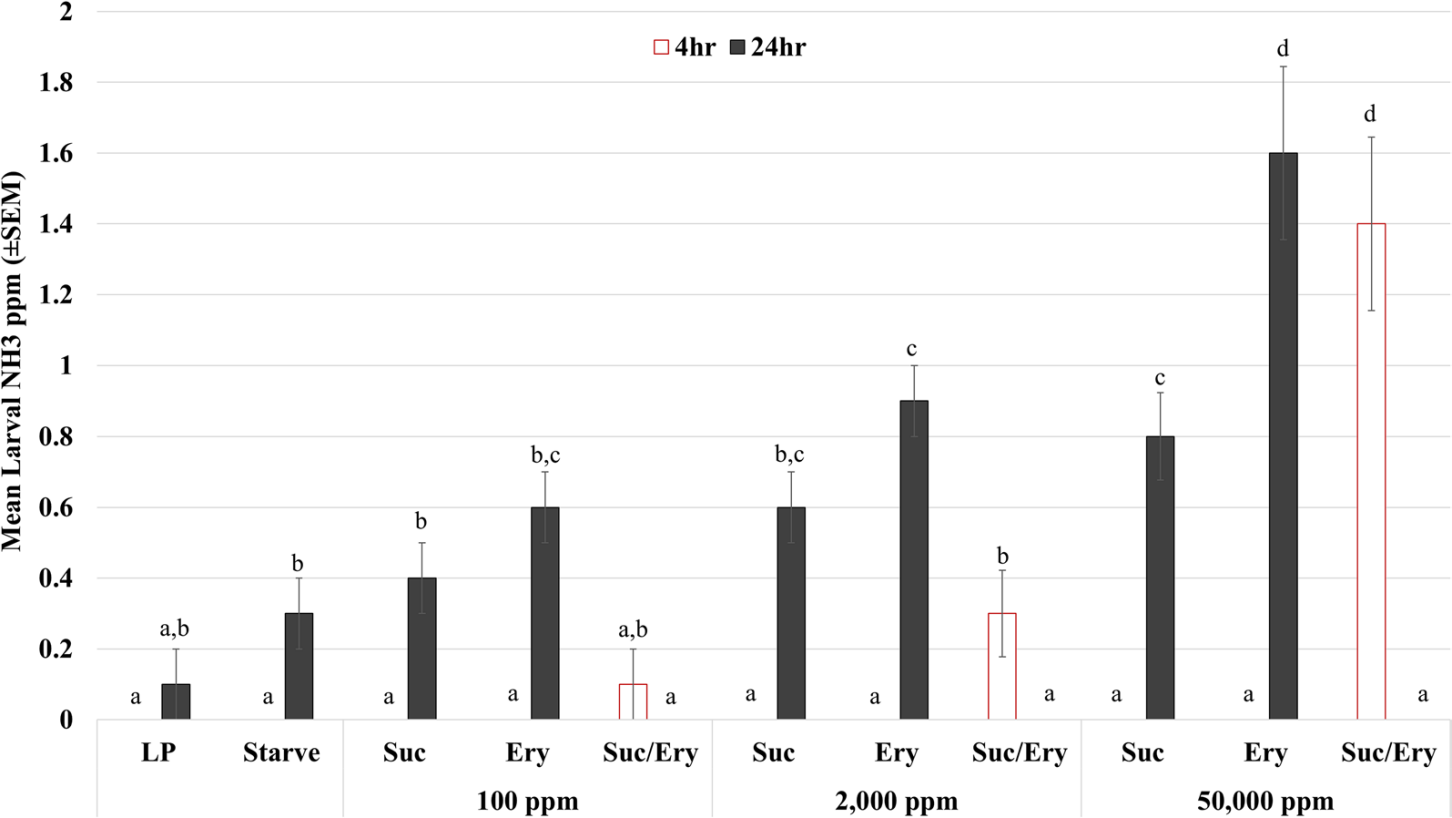
Larval mosquito excretion. Mean ammonia (NH_3_) detection in parts per million (ppm) from larval rearing water after third–fourth-instar *Aedes aegypti* larvae were exposed to increasing concentrations of sucrose (suc), erythritol (ery), or a combination. Control groups consisted of only liver powder food (LP) or a no-food starvation control (Starve). Water was analyzed at 4 h (light bars; *F*_8,44_ = 22.50, *P* < 0.0001) and 24 h (dark bars; *F*_8,44_ = 21.74, *P* < 0.0001). Bar graphs are represented with I-bars as standard error of the mean. Groups are annotated (a–d) for ANOVA/Tukey HSD with shared letters being insignificantly different

Treatments against larvae in all replicates of the negative control, sucrose, erythritol, or a 1:1 ratio of sucrose/erythritol, whether dosed at 25 mg (100 ppm), 500 mg (2000 ppm), or 12.5 g (50,000 ppm), resulted in no observed mortality after 72 h. Larvae were retained for further observation until they pupated and eclosed normally into adults. In excretion measurements, all of the nitrite and nitrate test samples came back negative after 4 h and 24 h. Ammonia, a key excretory product, fluctuated with treatments (Fig. 1). Only sucrose + erythritol blends yielded any detected ammonia after 4 h (*F*_8,44_ = 22.50, *P* < 0.0001), with 2000 ppm (*P* < 0.0001) and 50,000 ppm (*P* < 0.0001). After 24 h, erythritol-only treatments had the highest ammonia in ppm (*F*_8,44_ = 21.74, *P* < 0.0001) relative to all other treatments. As a trend, ammonia levels correlated positively with erythritol (*P* < 0.0159) and sucrose concentrations (*P* < 0.0004). Combination treatments with sucrose + erythritol at all concentrations yielded no detectable nitrogenous waste after 24 h (Fig. 1), but water was cloudy and became progressively more turbid across the 72 h of survival observation. Given the detection of ammonia at 4 h post treatment, we attribute the cloudiness to bacterial bloom after ammonia shock [22]. Erythritol did not show any effect on larval mosquitoes, aside from increased ammonia output.

**Fig. 2:**
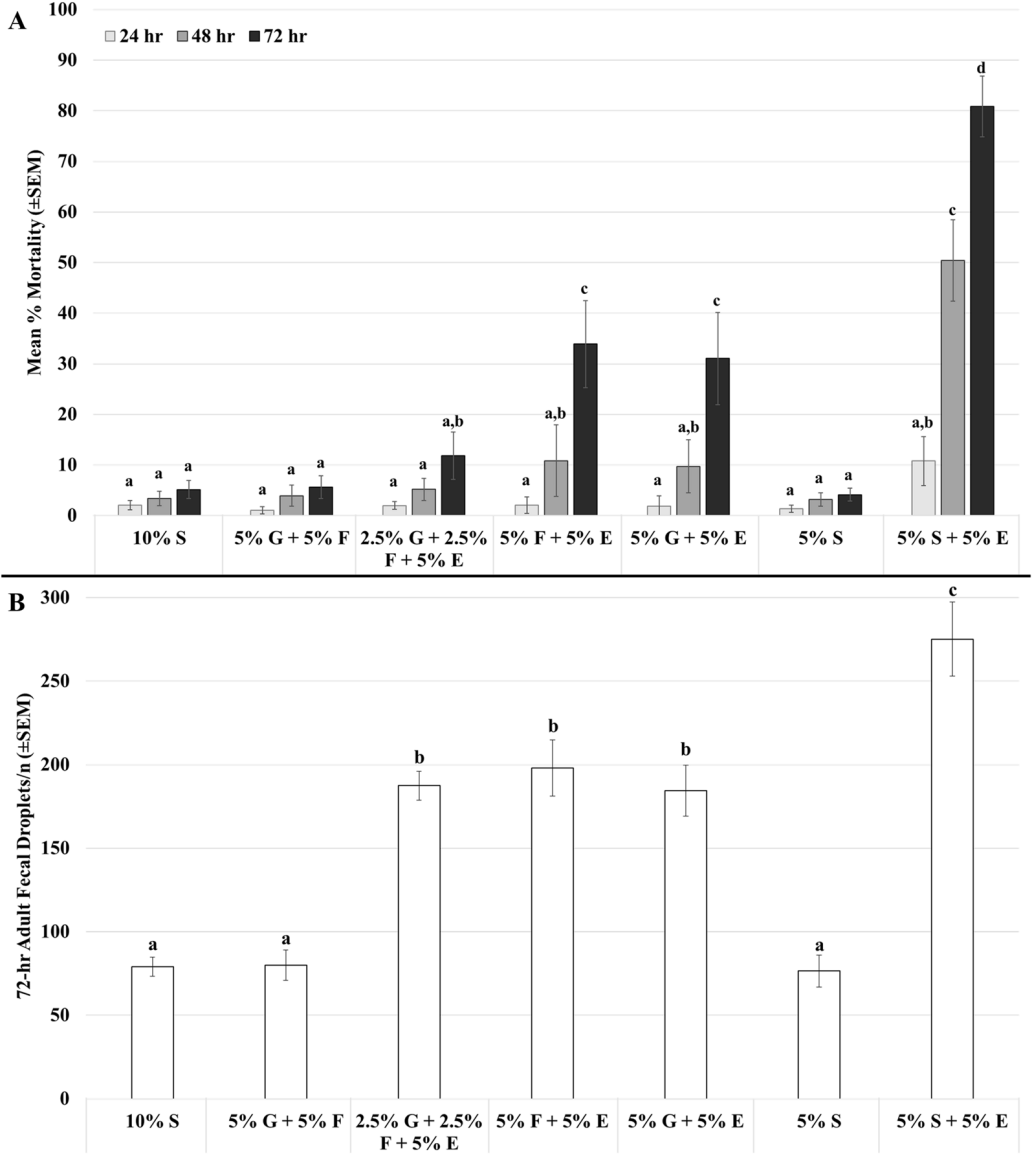
Adult mortality and excretion. **A** Mortality (*F*_2,273_ = 33.30, *P* < 0.0001) at 24, 48, and 72 h in adult *Aedes aegypti* following consumption of sucrose (S), glucose (G), fructose (F), and erythritol (E) combinations. **B** Fecal droplets adjusted for sample size (fecal/n) in adult *Aedes aegypti* 72 h after consumption (*F*_5,78_ = 26.80, *P* < 0.0001). Bar graphs are represented with I-bars as standard error of the mean. Groups are annotated (a–d) for repeated-measures ANOVA (mortality) or ANOVA/Tukey HSD (fecal), with shared letters being non-significantly different

In sugar combination bioassays, adult mosquito mortality increased significantly (Fig. 2A; *F*_2,273_ = 33.30, *P* < 0.0001) over the 3-day period for fructose (*F* = 15.46, *P* < 0.0002), glucose (*F* = 13.49, *P* < 0.0004), and sucrose (*F* = 79.58, *P* < 0.0001) combinations with erythritol. At 72 h, mortality was higher than other sugar combinations (*F* = 24.78, *P* < 0.0001). Fecal droplets, adjusted for the number of mosquitoes (n) in respective cups (Fig. 2B), were split into three significance groupings (*F*_5,78_ = 26.80, *P* < 0.0001). The non-erythritol sugar meals were equivalently lowest, all fructose/glucose/erythritol combinations were significantly higher than the previous, and sucrose with erythritol was significantly higher than the rest. Fecal droplets as a covariate were not significant to the 72 h mortality results (point estimate = −0.0079857, *z* = −1.556, *P* < 0.12), indicating that excretion was not correlated with the observed mortality.

**Fig. 3:**
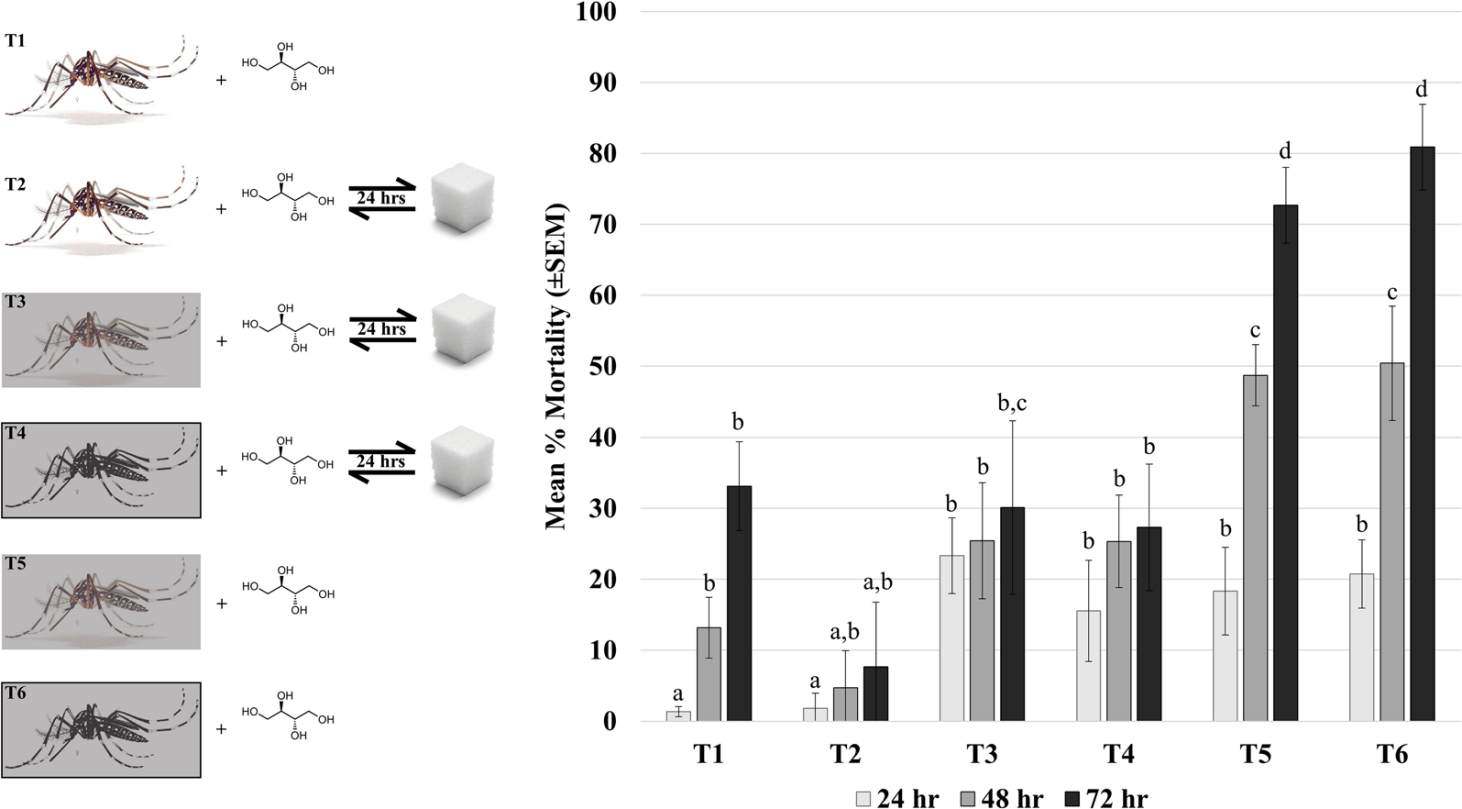
Adult starvation and recovery. Mortality in adult *Aedes aegypti* following consumption of 5% erythritol + 5% sucrose. Treatments were designated based on no prior starvation (T1, T2; unshaded mosquito), a slow starvation with 3% sucrose for 72 h (T3, T5; shaded mosquito), or complete starvation for 24 h (T4, T6; shaded and blackened mosquito). Half of the treatments allowed continuous access to the erythritol (T1, T5, T6), with the other half being swapped for 10% sucrose after 24 h to measure recovery (T2, T3, T4). Bar graphs are represented with I-bars as standard error of the mean. Groups are annotated (a–d) for repeated-measures ANOVA (*F*_2,197_ = 88.71, *P* < 0.0001), with shared letters being insignificantly different

For the starvation treatments, no significant difference was found for T1 at 24 h and T2 at all time points. Mortality was significantly higher (Fig. 3; *F*_2,197_ = 88.71, *P* < 0.0001) at 48 h for T1, all time points for T3 and T4, and the first 24 h of T5 and T6 but similar to each other. The mortality at 48 h for T5 and T6 was significantly higher (Fig. 3; *F*_2,197_ = 88.78, *P* < 0.0001) than previously mentioned groupings, while the mortality at 72 h for T5 and T6 was the highest (Fig. 3; *F*_2,197_ = 96.37, *P* < 0.0033).

**Fig. 4:**
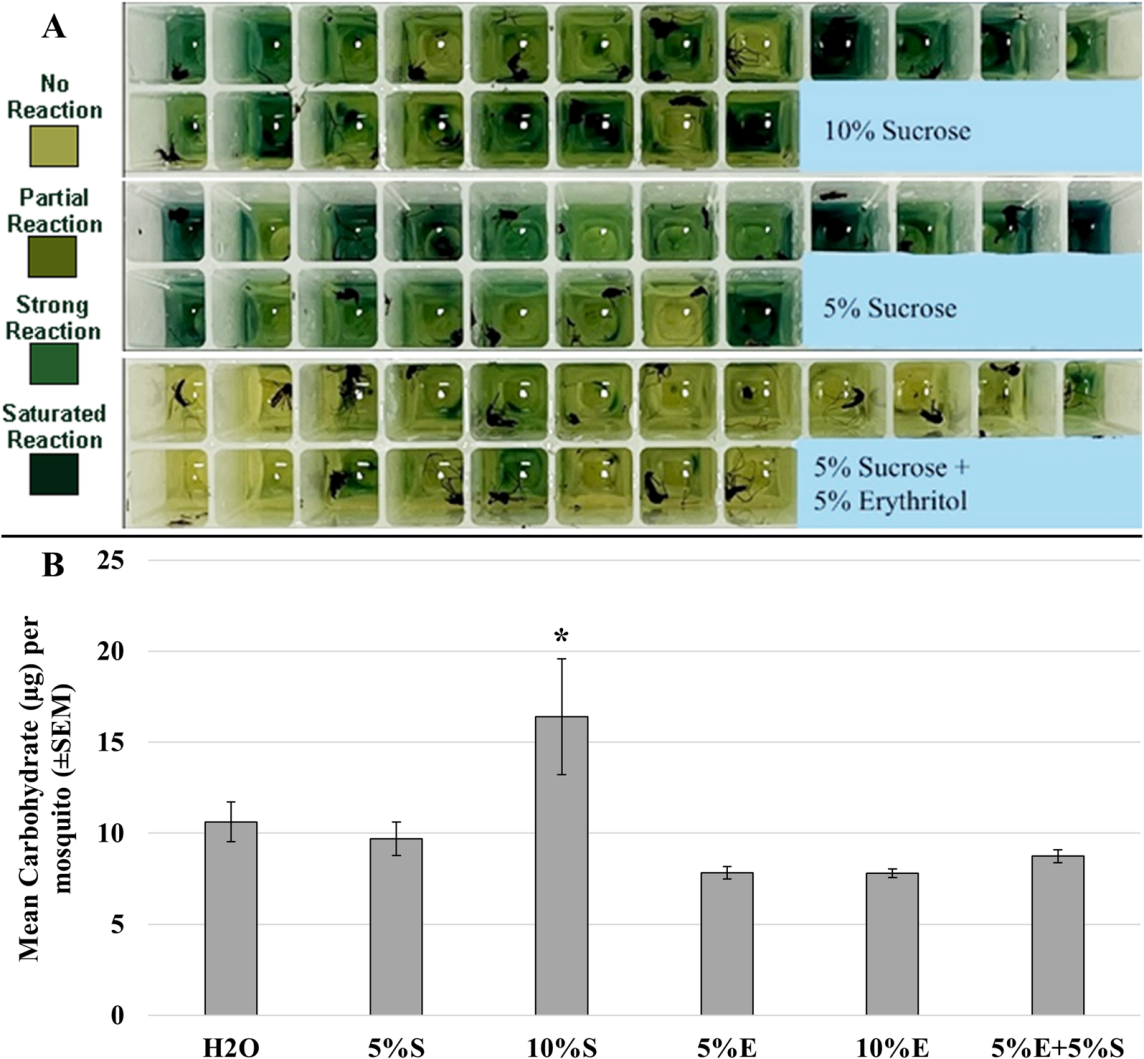
General carbohydrate measures. **A** Anthrone colorimetric reactions for demonstrating color saturation changes with respect to metabolized sucrose. **B** Carbohydrate estimates in adult mosquitoes fed water (H_2_O), 5% sucrose (5%S), 10% sucrose (10%S), 5% erythritol (5%E), 10% erythritol (10%E), and 5% erythritol + 5% sucrose (5%E + 5%S). Samples were homogenized in phosphate-buffered saline followed by centrifugation, and the supernatant was used on reaction plates in a spectrophotometer. Values per mosquito were estimated using the standard curve sucrose controls run on the same reaction plate. Bar graphs are represented with I-bars as standard error of the mean. Kruskal–Wallis and Wilcoxon signed-rank test (*H*_5,26_ = 14.1373, *P* < 0.0148) annotated with an asterisk

Estimates of what ingested sugars remained in the mosquito gut using anthrone reactions (Fig. 4A) were highest for 10% sucrose meals, with no statistical difference between other treatments (*H*_5,26_ = 14.1373, *P* < 0.0148). This indicates that, aside from 10% sucrose keeping mosquitoes well fed, the treatment groups did not exhibit an obvious reduction in available sugars after erythritol consumption (Fig. 4B).

**Fig. 5:**
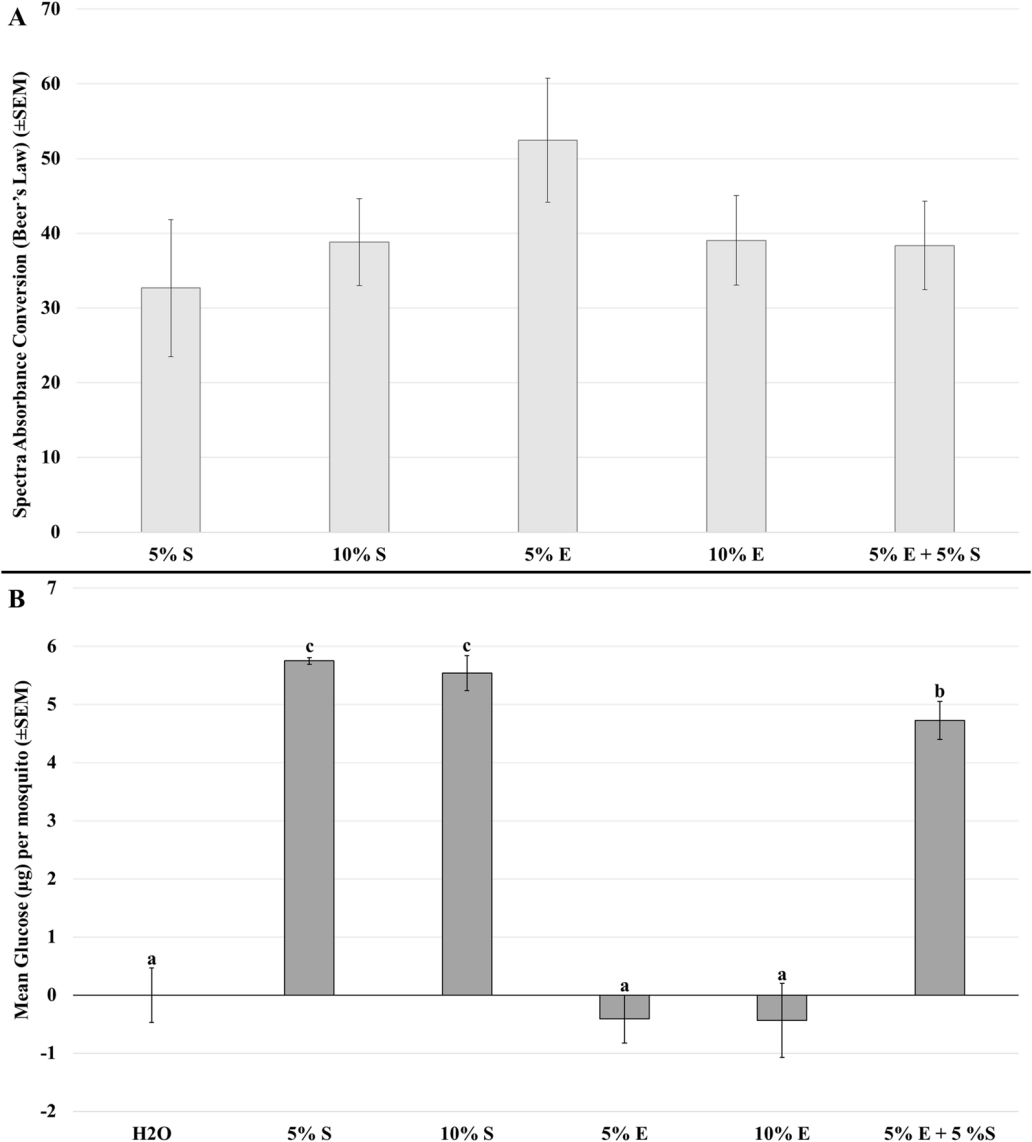
Enzyme and sugar measures. **A** Measures of α-glucosidase in adult mosquitoes fed 5% sucrose, 10% sucrose, 5% erythritol, 10% erythritol, and 5% erythritol + 5% sucrose. Spectra values were graphed with I-bars as standard error of the mean. Kruskal–Wallis with Wilcoxon signed-rank tests were not significant (*H*_5,35_ = 3.0432, *P* < 0.6933). **B** Free glucose measures in mosquitoes fed water (H2O), 5% sucrose (5%S), 10% sucrose (10%S), 5% erythritol (5%E), 10% erythritol (10%E), and 5% erythritol + 5% sucrose (5%E + 5%S). Glucose per mosquito was estimated using the standard curve glucose controls run on the same reaction plate. Bar graphs with I-bars as standard error of the mean. Groups annotated for Kruskal–Wallis and Wilcoxon signed-rank (*H*_5,24_ = 25.878, *P* < 0.0005) between treatment effects

After linear conversion with Beer's Law, the α-glucosidase reaction kits produce higher converted values with higher concentrations of α-glucosidase during the spectra measurements. The results were not significantly different between any of 5–10% sucrose or erythritol, or 5% erythritol + 5% sucrose (*H*_5,35_ = 3.0432, *P* < 0.6933). A visual trend was observed wherein 5% erythritol had nominally higher detected activity (Fig. 5A); however, it was apparent that actual attempted digestion via α-glucosidase was not different after erythritol consumption.

In contrast, free glucose measures were significantly different between treatments (*H*_5,24_ = 25.878, *P* < 0.0005). Three distinct groupings were observed (Fig. 5B), with both sucrose controls being equivalent (*P* < 0.027), the 5% erythritol + 5% sucrose treatment being lower (*P* < 0.047), and the water control, 5% erythritol, and 10% erythritol being equivalently the lowest in glucose yield (*P* < 0.016). The free glucose was a proxy for successful digestion of sucrose and supports the hypothesis that the mosquitoes fed only erythritol were starving, while the combination treatment of sucrose and erythritol demonstrated weak depression in the digestion as compared to plain sucrose meals at comparable concentrations.

## Discussion

Erythritol was ineffective against *Ae. aegypti* larvae, even at concentrations previously described to cause mortality [3]. However, adults consuming erythritol had inhibited glucose metabolism in *Ae. aegypti*. It appeared that α-glucosidase was expressed in response to erythritol, even if it is essentially non-nutritional. Glucose and fructose meals in adult mosquitoes do not require functioning α-glucosidase to provide energy and were essentially non-fatal in this study. This result is a circumstantial support of erythritol being a linear competitive inhibitor of α-glucosidase, similar to data obtained in true bugs [2].

If α-glucosidase inhibition causes excessive buildup of erythritol and undigested sugars, it would support osmotic damage, similar to having too much salt in the body, which has been observed in other flies [5, 12, 17]. Interestingly, aquaglyceroporin activity is both increased and loaded with erythritol in treated *Ae. aegypti* [23], suggesting that erythritol simultaneously increases excretion, regardless of any other functions [4, 6, 23]. The increased excretion may be collaterally purging nutritive sugars as well. Related outcomes were observed in vinegar flies where there was a low retention of sucrose as compared to erythritol [5], and increased levels of unmetabolized erythritol were detected in frass. Other flies experiencing this may have simply exhibited regurgitation [4, 6]. Although larvae did not die, they also excreted heavily, leading to increased ammonia waste within the water. This was especially true from the erythritol/sucrose combinations. Ammonia levels decreased after 24 h, but we believe this to be an indicator of system shock leading to bacterial de-nitrification in the bioassays [22]. This was evidenced most strongly by cloudy water from the sudden bacterial bloom, as observed by others [22]. In contrast, adult mosquitoes became prone to non-fatal diarrhea. We observed this in our study through increased fecal droplets but low mortality after glucose/fructose/erythritol treatments.

The anthrone tests, designed to gauge general sugar availability in the mosquito body, essentially implied that sucrose levels were equivalent across treatment groups. However, in the free glucose measurements, erythritol treatments did not contribute energy and may have even interfered with already available sugars. Functionally, this makes erythritol a starvation accelerant, leaving undigested sucrose in the mosquito. Free glucose levels were still elevated in mosquitoes ingesting the combination of erythritol and sucrose. In addition to not being α-glucosidase dependent, co-ingesting glucose and fructose improves energy yield [24], which may explain the low mortality in spite of diuresis in our bioassays. Alternatively, the mortality that was observed may have been due to slower energy gains when glucose and fructose were not ingested together [24], failing to prevent eventual starvation. It is most probable that erythritol, and perhaps α-glucosides inhibition in general, causes mortality in mosquitoes through malnutrition only.

Our data showed a lack of effect when mosquitoes were not previously starved, and reduced mortality among groups where treatments were removed after 24 h. Additionally, the anthrone tests support the idea that mosquitoes were laden with undigested sucrose, despite attempted metabolism with α-glucosidase. When combined with the involuntary excretion [4, 6, 23], erythritol may create a slowly degrading feedback loop that excretes all unmetabolized solutes [5, 12, 17, 25] and could explain the effectiveness of sucrose and erythritol together [3, 11]. Despite this and prior studies, it remains unclear whether the mannose-1-phosphate pathway is relevant, which was hypothesized to cause protein damage [9] or be upregulated to improve sugar metabolism [16, 26].

## Conclusions

The combination of evidence suggests that erythritol works by inevitably starving the mosquito, even if it takes the combination of diarrhea and low-calorie intake to succeed. Unfortunately, we do not believe that erythritol would overpower nutritional options for adult mosquitoes in the field, since erythritol is not competitively attractive over sucrose for mosquitoes and other flies [9, 25]. Unfortunately, erythritol is a low-potency toxicant in mosquitoes. Nutritional starvation in the field would be an impractical way of using erythritol against mosquitoes in vector control operations, as the overall effect is slow to accumulate and is offset by alternate competing food sources [25].

That is not to say there are no advantages to exploiting α-glucosidase as a target site, just that erythritol itself currently lacks compelling evidence as a main ingredient for mosquito and vector management. Mosquito larvae are affected, even if they survive, and their signs of toxicity imply there is still utility in the molecule. Erythritol can be a tool for physiological modeling, such as with the prior aquaglyceroporin findings [23]. Clever formulations could use erythritol as a supporting ingredient to create a low-toxicity cocktail of minor ingredients, similar to prior work using adulterated sugar blends as a base with other toxicants [11]. Erythritol is also water-soluble, and may allow for unique formulations as a supporting ingredient for solubilizing other non-water-soluble ingredients. For example, there are numerous plant-derived materials that can kill mosquitoes but do not yet have clear use patterns [27]. There is a global need for development of new insecticides, and although erythritol alone is not especially suitable, its use as a synergist or adjuvant should be explored. We would welcome more exploratory work with similar functioning molecules to find better candidate active ingredients in our global efforts against nuisance and insect vectors of disease.

## Data Availability

Datasets can be accessed from Salt Lake City Mosquito Abatement District or corresponding authors on request.
